# Integrated sorting, concentration and real time PCR based detection system for sensitive detection of microorganisms

**DOI:** 10.1038/srep03266

**Published:** 2013-11-20

**Authors:** Monalisha Nayak, Deepak Singh, Himanshu Singh, Rishi Kant, Ankur Gupta, Shashank Shekhar Pandey, Swarnasri Mandal, Gurunath Ramanathan, Shantanu Bhattacharya

**Affiliations:** 1 Department of Mechanical Engineering, Indian Institute of Technology Kanpur, India; 2 Department of Chemistry, Indian Institute of Technology Kanpur, India; 3 These authors contributed equally to this work.

## Abstract

The extremely low limit of detection (LOD) posed by global food and water safety standards necessitates the need to perform a rapid process of integrated detection with high specificity, sensitivity and repeatability. The work reported in this article shows a microchip platform which carries out an ensemble of protocols which are otherwise carried in a molecular biology laboratory to achieve the global safety standards. The various steps in the microchip include pre-concentration of specific microorganisms from samples and a highly specific real time molecular identification utilizing a q-PCR process. The microchip process utilizes a high sensitivity antibody based recognition and an electric field mediated capture enabling an overall low LOD. The whole process of counting, sorting and molecular identification is performed in less than 4 hours for highly dilute samples.

Highly sensitive, specific and real time detection of food and water borne pathogens is one of the major concerns of industry, homeland security, public health and clinical diagnostics internationally^1^. The United States Department of Agriculture (USDA) recommends zero tolerance of certain strains of bacteria, such as *Escherichia coli* O157:H7, *Salmonella typhi*, and *Listeria monocytogenes* in all food products. The rapid multiplication of bacterial cells make even very low concentration (< 10 cells) to be extremely harmful to human health^2^,^3^,^4^. A major aim of lab on chip technology development is to enable a reliable selective separation, counting and identification of these pathogenic bacteria in shortest possible time. In microchip based assays, the target microorganisms are often present in very low concentrations and thus making detection a major challenge. Detection using lab on chip strategies of bacterial cells and spores through micro-filters, fluorescence based immuno assays^5^,^6^, capillary electrophoretic methods^7^,^8^, combination of ultrasound standing waves (USWs) with optical waveguides^9^, variation over sensing surfaces^10^, micro-cantilevers/particle focusing and selection methods^10^,^11^,^12^,^13^, dielectrophoretic or magnetophoretic trapping concentration and sorting methods (DEP/MP)^14^,^15^,^16^,^17^,^18^ and immunological methods^19^,^20^,^21^,^22^ have been widely explored. In order to address the challenge given by extremely low concentrations of harmful biological species, the techniques corresponding to pre-concentration of samples formulate a viable strategy for detection. Dielectrophoresis based pre-concentration is highly compatible with micro-chip architectures and has been widely utilized for manipulating and sorting blood cells^23^,^24^, stem cells^25^,^26^, neurons^27^,^28^, bacteria and yeast^29^,^30^,^31^,^32^,^33^, DNA, Proteins^34^,^35^ etc. Further rapid DEP has been studied using optimized electrode design^36^ and in a electrodeless manner^37^,^38^. Recently another opto-electrokinetic manipulation technique combining an AC electric field with an optical illumination was employed to manipulate *S. oneidensis*^39^ although the capture and sorting in this work was performed on full grown concentrations and detection was only evaluated size-wise.

As regards detection of microorganisms the most robust ones are the immunological and nucleic acid based methods like PCR. While an immunological detection is not so highly specific compared to nucleic acid based test, it is fast and robust and can detect both the micro-organism and its toxin. However the immunological method has a limited utility due to lower sensitivity, poor binding affinity and interference effects posed by the contaminants (backgrounds)^40^ thus necessitating a PCR based identification. Therefore, a lot of biochips with PCR capabilities (nucleic acid based) have been around for quite sometime for enabling a robust identification of microorganisms like integrated gene analysis or by combining PCR and capillary electrophoresis^41^,^42^. Real time PCR of purified stx-1 (150 bp) of *E. coli* and other samples using TaqMan® PCR^43^,^44^,^45^,^46^ *etc*. Microchip technology for the sake of precision and accuracy must necessitate a combination of several methods. Research for such combinations of more than one step on a single microchip platform currently forms a new dimension of microchip technology. For example Neilsen *et al.*^47^ have successfully removed PCR inhibitors such as bovine haemoglobin and heparin from baker's yeast sample using on-chip dielectrophoresis and subsequent off-chip use of PCR techniques. Lagally *et al.*^48^ have shown a cell capture protocol using dielectrophoresis followed by hybridization based RNA detection wherein a total of sixty *E. coli* MC1061 cells can be detected. Recently Bhattacharya *et al.*^33^ have shown an integrated platform with dielectrophoretic concentration of *L. monocytogenes* V-7 and its end point PCR detection using SYBR-Green PCR^33^ where a detection limit of around 60 cells from a sample of concentration 10^4^ cfu/ml. One of the major issues that need to be evaluated in the different biochips as discussed is to develop a methodology wherein complete cell sorting sample pre-concentration, immunological recognition and nucleic acid based detection is carried out in one single platform.

In this work, we report a completely novel detection methodology where metallic nano-particles are used to mediate immuno-conjugation of targeted microorganisms, which are further sorted and concentrated through DEP technique and detected through nucleic acid based method (q-PCR) in a single microchip. All diagnostics are performed within a pico-liter micro-channel by use of optimized inter-digitated microelectrodes and a micro-scale thermal cycling mechanism. This is schematically represented in Supplementary Fig. S1(a). The advantage offered by this architecture over contemporary detection platforms is the high specificity of recognition and quantitation of the target microorganisms at a limit of detection (LOD) of 10^2^ cfu/ml with about 10 bacterial cells inside the micro-chip with a total assay time of less than 4 hours at LOD. The technique developed through this work couples high selectivity of immuno-recognition (sorting), active capture (DEP) and high specificity of q-PCR.

## Results

### Nano-conjugated *E. coli* DH5α cells

The nano-conjugation was primarily achieved by using a gold nanoparticle which was coated with a secondary antibody (Goat anti-mouse IgG). A primary anti *E. coli* antibody was used to capture the *E. coli* DH5α cells and bind this to each other through the nano-particle bridges. So, the binding mechanism was multiple cells binding to each other through many secondary antibody coated nanoparticles linked up with primary antibodies binding in turn to the target micro-organisms. This mechanism has been schematically illustrated (see Supplementary Fig. S3). SEM micrographs of vacuum dried samples of normal and nano conjugated bacterial cells is shown as Supplementary Fig. S4a and S4b. The EDAX spectrum performed on the conjugates shows a strong presence of gold (see Supplementary Fig. S 5).

### Dielectrophoretic capture of bacterial cells on interdigitated microelectrodes (IDMEs)

To begin the process, bacterial solutions with normal cells were injected by a micro syringe pump at a continuous flow of 20 μL per hrs) and an A.C. signal of magnitude 10 volt peak to peak (V_pp_) with a sinusoidal nature was applied at 10 kHz frequency to the interdigitated electrodes. The bacterial cells injected into the biochip were captured on the interdigitated electrodes. Images of this capture process were acquired after every 3 minutes using a Nikon microscope mounted with a CCD camera (Eclipse, 80i) and subsequently the fluorescence signal was computed in real time and plotted. Following this, the DEP based capture of nano-conjugated *E. coli* cells were also carried out. A shift was observed in the capture frequency of the conjugated cells from 10 kHz (in case of normal bacterial cells without any conjugation) to 650 kHz. The capture voltage was the same 10 V_pp_ as used earlier. Figure 2 (a)–(d) show the representative images of the capture process. As the electric field is turned on the fluorescently labelled microorganisms (both conjugated and unconjugated) get captured on the electrodes. The master data set is built by acquiring one data point every 3 minutes as the capturing proceeds. The average fluorescence data is computed over time by looking at the variability of the master data set and limiting such variability to a fixed range (see Supplementary Fig. S3). Figure 2 (e) and (f) is a consolidated summary of fluorescence growth generated from the master dataset with respect to time as the capture process happens at different inlet concentrations for both type of cells as discussed above. The fluorescence intensity grows exponentially followed by a plateauing towards the end signifying substantial capture towards the beginning followed by a saturation owing to maximum entrapment of the whole sample. Fluorescence signal touches a maximum value and plateaus off in time owing to the setting up of a dynamic equilibrium in the no. of capture sites thereby meaning that any occupied site is reoccupied with a fresh particle at the behest of the already existing particle. Electro-thermal flow occurs due to joule heating coupled to illumination (provided by the fluorescence microscope) in the solution which limits the bacterial cell capture resulting in plateauing of the fluorescent signal^49^,^50^. We have observed an identical behaviour for capture of fluorescent micro-beads^51^. Also, the exponential growth of the signal which happens immediately as the flow is introduced is proportional to the inlet concentration and we have been able to observe a clear difference corresponding to 10^9^, 10^7^, 10^6^, 10^4,^ 10^3^ and 10^2^ cfu/mL. For the least inlet concentration the growth in signal is registered after around 200 minutes owing to the extreme lean nature of the initial concentration of bacterial cells which coupled by a low injection rate of only 20 μL/hour accounts for this delay. The low flow rate of 20 μL/hour is necessary to obtain high yield of the capture process.

### Capture efficiency studies of DEP

The capture process on the biochip was validated through plate culture and counting technique and by using Malassez slide^52^. For plate counting, original solutions of *E. coli* DH5α cells with concentrations in the range from 10^9^ cfu/mL to 10^7^ cfu/mL were passed through the micro channel – IDME assembly and captured over the IDMEs. The injected sample after passing over the IDMEs was collected at the outlet of the biochip and these flow through were further diluted and plated. The various inlet solutions were also diluted to the same order as the outlet. The pre and post DEP solutions serially diluted for 5 and 4 times respectively were spread on LB agar plates containing ampicillin (100 μg/mL) and incubated for about 24 hrs at 37°C. The pre-DEP sample plates showed counts of colonies upto an extent of an order of magnitude more than the post-DEP samples. Supplementary Fig. S6 shows these images and the counts in a tabulated manner. This also indicates that the cells are still viable after getting influenced by the applied electric fields. The images of the culture plates were acquired on a gel documentation system (M/s BioRad) and number of bacterial colonies counted using Image-J (NIH). The average capture efficiency of DEP for the various samples was obtained as 70–75%. This was further validated by counting the cells using Malassez slides^52^. The counting data are presented in Table 1. The captured cells are not affected by any irreversible damage during the DEP experiment^53^.

### RT- PCR of bacterial cells on the biochip

Once the desired capture of microorganisms was accomplished in the bio-chip the identification step through RT- PCR was initiated. We first performed RT-PCR with normal *E. coli* DH5α cells captured over the IDMEs in different concentrations viz. 10^9^, 10^7^, 10^6^, 10^4^, 10^3^ and 10^2^ cfu/mL. The thermal cycling was executed on the micro-chip (See supplementary Fig. S1). During the PCR the fluorescence imaging was performed at 10 frames per second (FPS) and a digital video of the whole process was acquired. All frames those corresponding to the end of the 72°C step were separated and binned together as a function of the no. of cycles. Some representative frames corresponding to cycle no.'s 1, 10, 20 and 25 for initial pre-DEP inlet concentrations 10^9^, 10^7^, 10^4^, 10^3^ and 10^2^ cfu/mL are illustrated (see Supplementary Fig. S7). The intensity of all these frames has been determined by image-J using the pixel count method. Further the relative fluorescence intensity has been computed between the corresponding frame of a certain cycle no. with respect to that of the initial fluorescence intensity value and these ratios have been plotted on logarithmic scale w.r.t the corresponding cycle no.

Figure 3 illustrates the cumulative cycle wise logarithmic ratio plot of the fluorescence intensity in which each initial concentration comprises of a linear, exponential and plateauing part in overall signal which is similar to the real time data obtained in any RT-PCR machine. The distinction of the initial template concentration which is signified by the change of the linear to exponential characteristics of the acquired signal referred hereinafter as transition point.

The linear to exponential signal growth is registered within the first 3 cycles for concentrations 10^9^ and 10^7^ cfu/mL whereas this change over starts at 10^th^ cycle for lower concentration corresponding to 10^4^ and 10^3^ cfu/mL and at 23^rd^ cycle for 10^2^ cfu/mL. Further there is a marked distinction in the transition point for 10^4^,10^3^ and 10^2^ cfu/mL, as template concentrations are already quite low. A similar behaviour is observed in the conjugated samples.

### Specificity test

The conjugated *E. coli* cells were mixed with *Paracoccus* cells (conc. 10^5^ cfu/mL) in equal proportions by volume for testing the specificity of detection of this assay. As observed before, the DEP process was able to capture 75% of the target biological entities (normal or conjugated cells) and the uncaptured retentate output of the chip contained a few (25%) of the total no. of biological entities flown in. (Supplementary Fig. S8). We have passed the mixture containing the conjugated *E. coli* DH5α and *Paracoccus* cells and performed the DEP base capture at a capture frequency 650 KHz (corresponding to the signature frequency of the conjugates). The retentate solution coming out of the biochip was injected inside two new channels in the same biochip and RT-PCR was performed on one channel using the primers for conjugated *E. coli* DH5α and in the other channel with primers specific to *Paracoccus* strain. Four such biochips were used where in the first the retentate solution coming out was investigated for RT-PCR after 1 pass, 2 passes, 3 passes and 4 passes respectively. The conjugated *E. coli* DH5α was captured and thus its concentration after each pass would fall in the retentate whereas the uncaptured *Paracoccus* would have similar concentrations. The maximum fluorescence intensity for *Paracoccus* seems to be unchanged in all the 4 biochips whereas that of the conjugated *E. coli* DH5α goes down (also plotted separately as Figure 4) which shows highly specific capture of the conjugates in our biochip. This followed with RT-PCR which adds on to the selectivity and makes our chip highly specific to the recognition of various microorganisms. Trials have also been carried in Real fruit juice samples in order to ascertain the specificity of our biochip while reading real samples. The fruit juice solution was diluted (2 fold) using DI water and the washed bacterial cell pellets were suspended in the diluted fruit juice. The DEP based detection was achieved by the process described earlier. Cells got captured at a frequency of 10 kHz and our biochip showed a fluorescence response and also sensitivity to q-PCR. (Supplementary figure S9–11).

## Discussion

Deployment of chip based detection techniques has distinct advantages in order to enhance the detection speed, miniaturization and sensitivity. The main challenges in developing a lab on chip device are an overall integration of several sub components in a single microchip for carrying out different recognition/detection protocols. Such an integrated approach should be specific, sensitive, rapid and lower minimum detection limit. The device presented in this work is completely integrated and does sensitive (around 8 cells in a volume of 80 μL) and specific recognition and counting of microorganisms with high accuracy in less than 3 hours time. Compared to several conventional methods for detection of microorganisms, which sometimes may need several hours to days on a lab scale, our biochip can perform this integrated detection in very short amount of time and still detect microorganisms with the same foolproof methodology. Bhattacharya *et al.*^33^ reported about integration but they had detected the amplification at the end point of the PCR cycle whereas here real time monitoring is possible with this system. Kwon *et al.*^39^, 2012 reported about separation of bacterial cells by rapid electrokinetic patterning using focused Laser beam. But this system is a closed system where bacterial samples are injected and separated and valid at higher bacterial concentration. For very dilute samples one needs to concentrate it; so continuous flow type system is required which is under process of development as reported by Kwon et al. Our system already offers this feature. Further it requires an extra source of Laser light for detection. Report by Kwon *et al.*^39^ is silent about integration of PCR on the same chip. The biochip described in this paper is designed as a diagnostic tool for meeting some major detection challenges in the food, water and pharmaceutical industry.

## Methods

### Device design and fabrication

The biochip has been developed in a hybrid platform with a lower die made up of silicon with imprinted micro-electrode array (IDME) and an upper die made up of replicated (Poly dimethyl siloxane) PDMS^51^.

### Realization of the upper die

**Figure 1:**
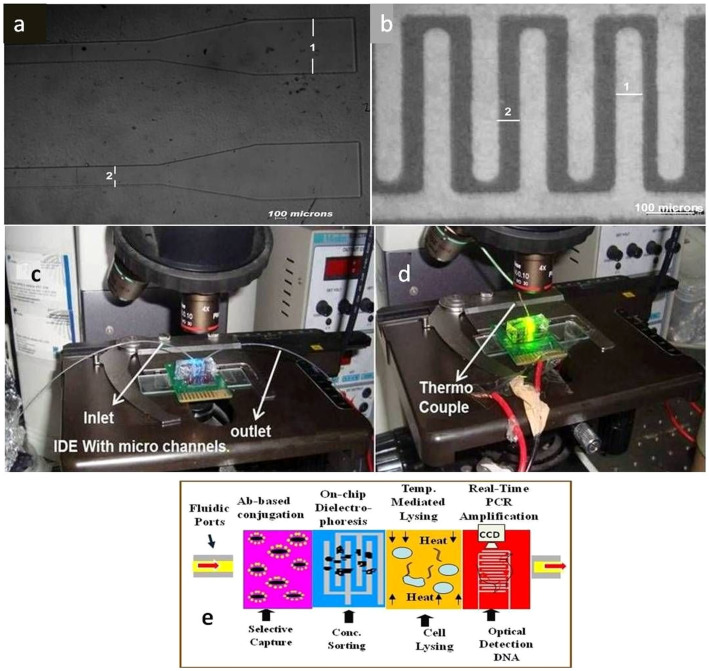
(a) Image of micro-channel, (1) dimension of inlet/outlet ports is around 650 μm and (2) the main portion have dimension around 225 μm, (b) Images of interdigitated electrodes (1) individual width around 53.562 μm and (2) inter electrode spacing of 42.322 μm (c) Image of dielectrophoretic capture (d) Image of RT-PCR based detection (e) Schematic of the sequential events within the Biochip.

**Figure 2:**
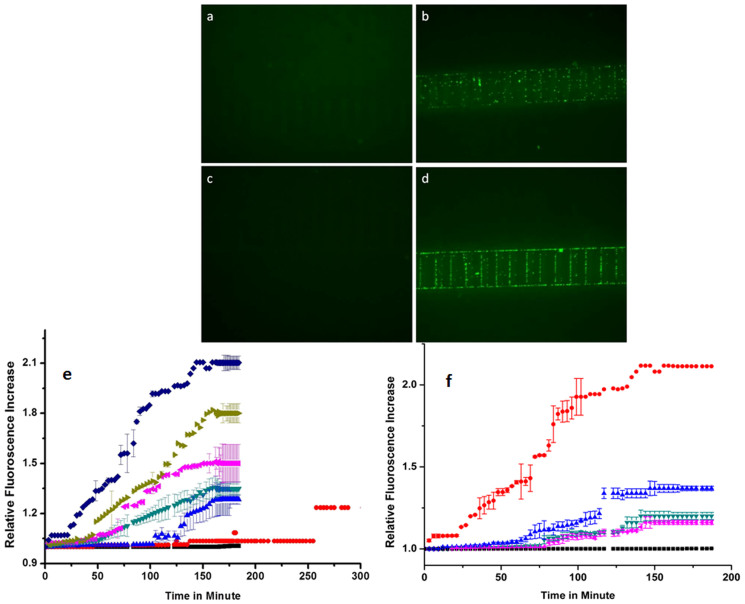
(a), (b) Real time snap of captured normal cells at 10 KHz frequency, (c), (d) Real time snap of captured conjugated cells at 650 KHz frequency (e) Plot showing the trend in increase in fluorescence intensity during DEP of different bacterial cell (normal) concentrations with respect to time, 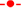: 10^2^ cfu/mL; 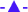: 10^3^ cfu/mL; 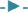: 10^4^ cfu/mL; 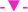: 10^6^ cfu/mL; 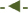: 10^7^ cfu/mL; 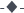: 10^9^ cfu/mL; 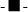: control, (f) Plot showing the trend in increase in fluorescence intensity during DEP of different bacterial cell (conjugated) concentrations with respect to time, 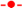: 10^9^ cfu/mL; 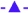:10^6^ cfu/mL; 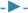: 10^4^ cfu/mL; 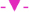: 10^3^ cfu/mL; 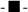: control.

**Figure 3:**
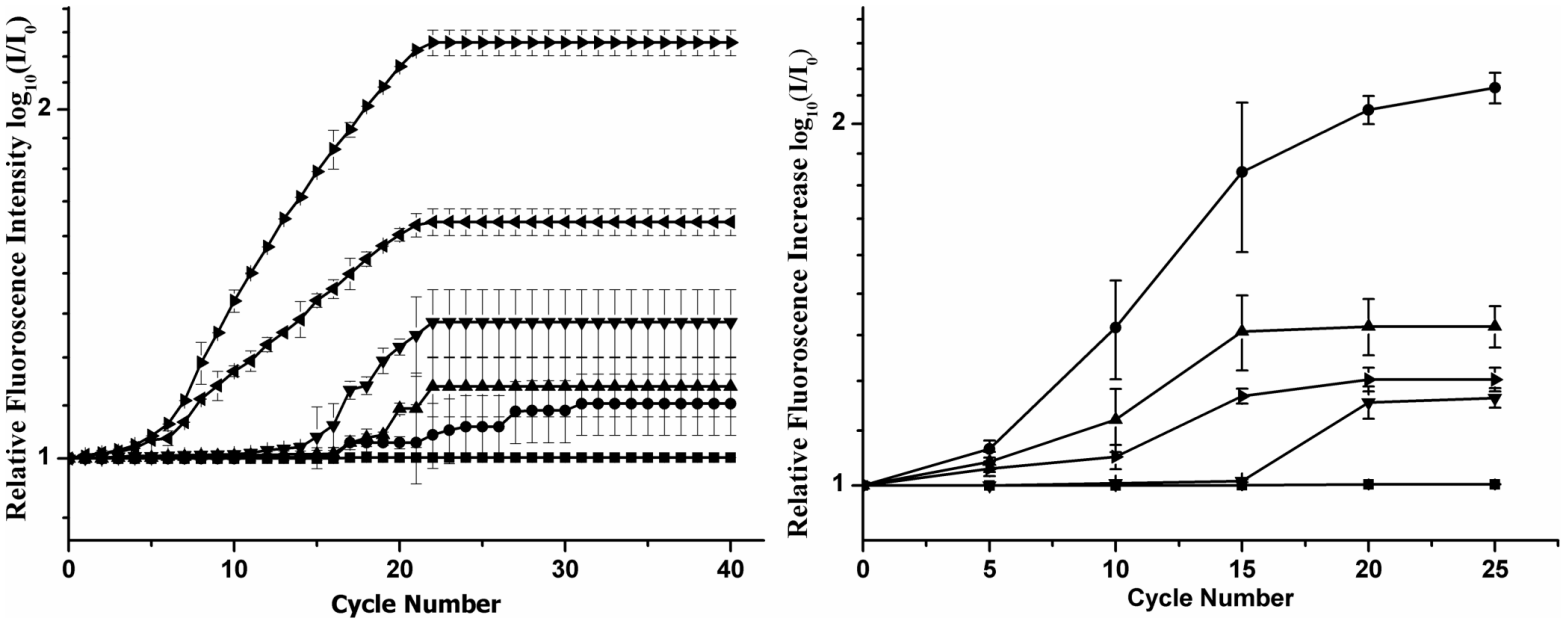
(a) Plots depicting the trend in fluorescence increase during RT PCR with time for normal cells. 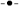: 10^2^ cfu/mL; 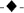: 10^3^ cfu/mL; 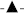: 10^4^ cfu/mL; 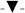: 10^7^ cfu/mL; 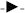: 10^9^ cfu/mL; 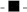: control, (b) Plots depicting the trend in fluorescence increase during RT PCR with time for conjugated cells. 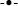: 10^9^ cfu/mL; 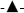: 10^6^ cfu/mL; 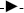: 10^4^ cfu/mL; 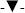: 10^3^ cfu/mL; 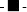: control.

**Figure 4:**
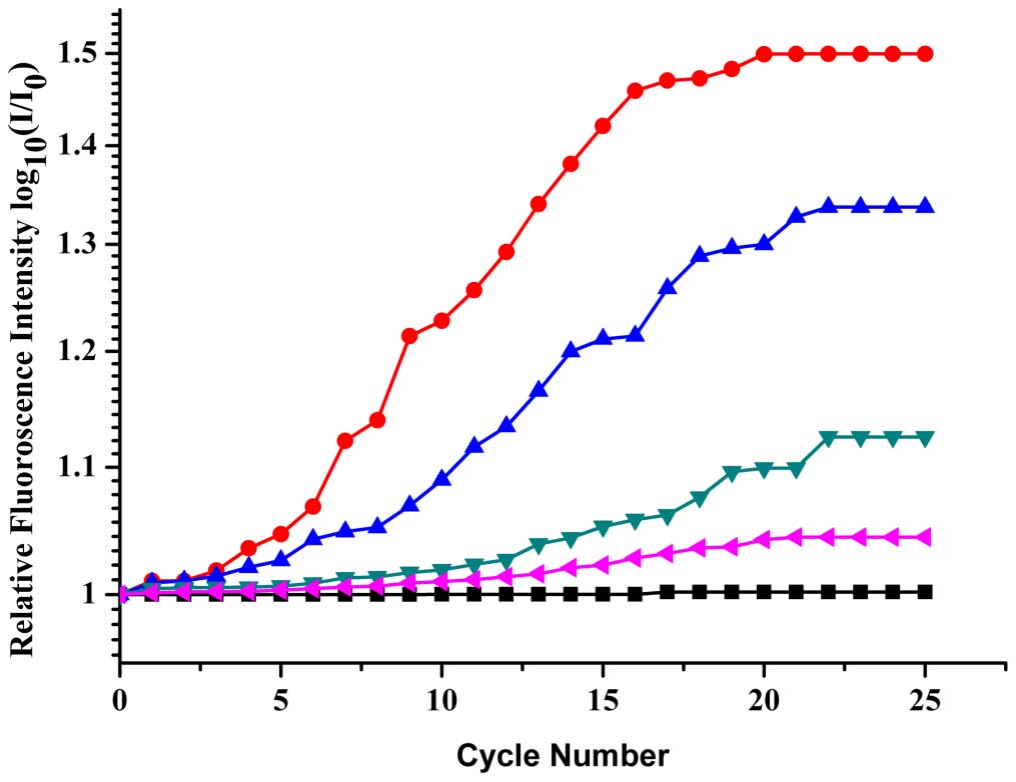
Reduction of RT-PCR signals of *E.coli* DH5α (conjugated, concentration 10^5^ cfu/mL) in the flow through solution with each pass, 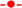: one pass; 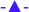: second pass; 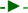: third pass; 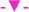: fourth pass; 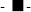: control.

Micro-channels were fabricated in a soft polymeric matrix by soft lithography process followed by replica moulding technique^54^,^55^,^56^. A mold was made using SU8 2007 with 10 microns feature size which was treated with mold release agent hexamethyldisilazane (supelco analytical, USA) for 30 minutes and PDMS was replicated over these using a 10:1 ratio of silicone rubber and curing agent (Sylgard 184, Dow Cownig, Midland, USA). Figure 1a shows a photograph of micro-channel taken under an optical microscope at 10 × magnification.

### Realization of the lower die

The lower die was fabricated in silicon by photo-lithography and lift off process using S-1813 positive tone photoresist (M/s Shipley) followed by metal sputtering wherein a layer of 20 nm chromium and 200 nm nickel was deposited and lift off process was carried out. Figure 1b shows the microscopic images of a set of fabricated electrodes with an inter electrode spacing of 42.322 μm and the individual width around 53.562 μm.

### Assembly of upper and the lower dies

The micro-channels were mounted over the lower die, after sputter coating the electrodes with Silicon dioxide (200 nm) and alignment of the geometrical axes of both channels and electrodes were carried out under a microscope. Prior to this mounting and alignment both the oxide and PDMS surfaces were exposed to oxygen plasma using a Harrick Plasma tool (115 V, 60 sec) and the PDMS die was punctured with a syringe needle in the inlet/outlet ports and this was used later for injecting the sample solution into the microchannel. An extra hole was drilled in the PDMS outside the microchannel boundary to monitor and control the biochip temperature as it was required for inserting the thermocouple.

### Packaging & Instrumentation

The PDMS – Silicon hybrid assembly was pasted on a custom designed printed circuit board (PCB) with thermally conductive epoxy. The PCB contained an embedded heater and also gold-plated bond pads that were used to electrically connect the biochip to the PCB by wire-bonding using aluminium (10 micron diameter) wire (West Bond, CA). The PCB was further electrically connected to a function generator (33220A, 20 MHz Function/Arbitrary Waveform Generator, Agilent Technologies, USA) for providing the AC frequency and to a NI-PXI-1031 (National Instruments, USA) controller box equipped with a programmable power supply and a digital multimeter. The NI-PXI-1031 module has been used for measuring and controlling the temperature of the biochip during the execution of the PCR cycle. A K-type thermo couple was inserted into the hole drilled outside the microchannel in the PDMS half by using a glass sleeve and hysol epoxy. The sensing tip of the thermocouple was coated with a thin layer of thermal epoxy to ensure a uniformity of temperature readout. An offchip syringe pump (11 PLUS, Harvard Apparatus, USA) was used to flow the sample solutions with different concentration of microorganisms. The entire process of flow, capture and PCR was performed on the optical stage of an epifluorescence Nikon80i microscope, equipped with a Pixera CCD camera. (Figure 1 c, d).

### Bacterial strain used in this study

*E. coli* DH5α cells used in this study were pre-transformed with TA cloning vector (purchased from Bangalore Genei) having a reductase gene (mntAa-987 bp, Genbank accession no. KC691252) as a DNA insert^57^,^58^. *E. coli* DH5α cells were grown in LB (Luria-Bertani, M/S BangloreGenei) culture medium containing 100 μg/mL of ampicillin at 37°C overnight in a shaking incubator (Mahendra Scientifics, India). Growth was detected by measuring the optical density (OD_600_) of the growth medium and an OD_600_ of about 2.5 was estimated to be reflective of late exponential growth phase. *Paracoccous* sp. strain DMF has capabilitiy to degrade dimethylformamide(DMF), dimethylamine. This strain has dimethylformamidase (DMFase) gene on a plasmid which converts DMF to dimethyl amine. Partial amplification of DMFase gene (~1 kb) was used here for identification of this strain. *Paracoccus* cells were grown in minimal media upto late log phase as described by Swaroop *et al*^59^, harvested by centrifugation and resuspended in PBS. Rest of the procedures was same as as described above for *E. coli*.

### Sample preparation for performing cell capture and counting using DEP

Bacterial cells were harvested from the culture medium by centrifugation at 5000 g and washed several times with 1 × PBS buffer and finally resuspended in DI water. About 1 mL of this suspension in DI water contained 10^9^ cfu/mL as estimated by plate count methods. Serial dilution of this initial sample was carried out using deionized water till a titre of 10^2^ cfu/mL was obtained. Subsequently the bacterial cells were dyed using acrydine orange (excitation maxima at 502 nm and emission maxima at 525 nm, concentration: 3 μg/mL). For preparation of bacteria spiked fruit (***Litchi chinensis***) juice samples, cells were collected, washed and resuspended in a two fold dilution of the commercially obtained samples. (M/S Real, Batch No.:MW0266, Date: 25/07/2013).

### Preparation of nano-conjugated *E. coli* DH5α cells

A part of the culture solution containing 10^9^ cfu/mL of microorganisms was prepared by the procedure described above and mixed with anti *E. coli* antibody solution (0.1 mg/mL) (M/S Abcam ab25823). This mixture was incubated for 15 minutes at 37°C and goat anti-mouse IgG (heavy and light chain) coated with 10 nm gold particles (Ted Pella, Inc., Redding, CA, USA) was added. The final solution was again diluted 50 fold and incubated for 10–15 min. The resulting solution was vacuum dried onto the stage of a scanning electron microscope (SEM) and micrographs were obtained. Figure S4 shows the SEM micrographs of the normal cells and the conjugated cells respectively.

### Sample Preparation for RT-PCR

*E. coli* DH5α cells were grown harvested and washed as reported above. Separate samples with concentrations – 10^9^, 10^7^, 10^6^, 10^5^, 10^4^ and 10^2^ cfu/mL were prepared in DI water. AAF1forward (5′ATGGAACTGGTAGTAGAACCCCTC3′) and AAR2 reverse (5′TCAGACGCCGCTGGGATAGAACGC3′) primers were used to amplify the DNA fragment of size ~ 1 Kb of *E. coli* DH5α^57^,^58^. The PCR was carried out on the biochip using a q PCR kit with SYBR green detection (M/S Qiagen Inc.) wherein PCR mix was prepared by mixing 2 × buffer, both forward and reverse primer and water respectively and subsequently injected into the biochip with captured target and thermally cycled.

### Dielectrophoretic capture of bacterial cells on the biochip

The dielectrophoretic capture of bacterial cells has been achieved by continuously flowing the bacterial cell solutions via a microsyringe pump. The IDMEs were provided with an A.C. signal at 10 kHz frequency (for normal bacterial samples) and 650 kHz frequency (for conjugated bacterial samples) by using a function generator (33220A, 20 MHz Function/Arbitrary Waveform Generator, Agilent Technologies, USA). The fluoroscence labelled bacterial cells were got captured over the IDMEs. The images of the captured cells were obtained by using an inverted Nikon microscope mounted with a CCD camera (Eclipse, 80 i) and hence the fluorescence signal was analyzed in real time and plotted.

### Characterization with inverted fluorescence microscope

An inverted fluorescence microscope (Nikon ECLIPSE 80 i, Japan) was used to visualize and characterize qualitatively and quantitatively the cell capture and the PCR process inside the biochip. The microscope was equipped with tetramethylrhodamineisothiocyanate (TRITC) filter (excitation wavelength 540–565 nm, dichroic cut-off wavelength 565 nm, and emission wavelength 605–660 nm) enabling the monitoring of fluorescent dyes viz., acrydine orange used for DEP based counting and SYBR green used for RT-PCR detection. A CCD (charged coupled device) camera (Media Cybernetics SN: Q20656) mounted on the eyepiece of the microscope would rapidly capture fluorescence images of the micro-channels at regular time intervals. The samples of various concentrations were injected into the microchannels and DEP capture was performed for 45 minutes to 4 hours depending on the richness of the mixture. The images were processed using ImageJ (NIH) and the spatial value of fluorescence over the capture electrodes (present throughout the length of the microchannel excluding the inlet/outlet ports) were obtained for the various times of capture. The mean signal intensity was calculated corresponding to the maximum time point. The same setup was used to capture the images of the micro-channel during the thermal cycling process (PCR stage) corresponding to the end of the 72°C step of each PCR cycle. The growth in fluorescence signal with the progress of the PCR reaction was processed in an identical manner and the logarithmic ratio between the intensity of the images at the end of every 72°C step and the initial intensity of the chamber before the start of the PCR cycle was plotted with respect to the cycle number.

### Thermal cycling on the biochip

The thermal cycling has been carried out on the Biochip with an instrumentation strategy as indicated below. The temperature monitoring on the biochip was performed through the thermocouple and contact was maintained between the sensing tip and silicon base. The embedded heaters on the PCB as described earlier were connected to the output of the programmable power supply, NI-PXI-4110 box (National Instruments, USA) and helped in ramping up the surface temperature inside the chamber containing the PCR mix while the readout of the biochip temperature was acquired by the thermocouple sensor into a digital multi-meter device assembled in the same PXI box. A proportional-integral-derivative (PID) controller was programmed in Labview version 9 (National Instruments) to synchronize the thermal cycling on the biochip. Supplementary Fig. S1 a, b, c show the schematic of the instrumentation for performing thermal cycling and the real signal plot of the thermal cycle as extracted by the Labview. The ramp up rates obtained were around 3.1°C/s and the cooling rates obtained by keeping the biochip assembly over an ice box comes out as 2.1°C/s using PID control.

The PCR program that was used comprised of initial temperature of 95°C for 5 min (to promote cell thermal lysis) followed by 40 cycles of 95°C (for 10 secs) for denaturation, 55°C (for 5 secs) annealing and 72°C (for 15 secs) extension.

## Author Contributions

M.N. did overall onchip experimentations and integration, D.S. did all off-chip validation and experimentation, R.K. performed DEP based capture on chip, H.S. did labview code for thermal cycling, A.G. did chip design and fabrication, SSP developed the image analysis for onchip PCR fluorescence measurements, S.M. did all the nano-conjugation work. All figures and supplementary figures were jointly prepared by all authors. All work has been carried out and engineering conceived under the joint Supervision of S.B. and G.R. and the manuscript has been written by them jointly with inputs from all other authors.

## Supplementary Material

Supplementary Information Supplementary Information for the work

## Figures and Tables

**Table 1:** Cell counting using Malassez Slide

Cell concentration (cfu/mL)	Pre DEP	Post DEP	% of capture Efficiency
10^9^	9982	2495	75
10^7^	9985	2497	74.9
10^6^	9965	2600	73.9
