# Supplementary material for: Integrated sorting, concentration and real time PCR based detection system for sensitive detection of microorganisms
Source: Sci Rep. 2013 Nov 20;3:3266. doi: 10.1038/srep03266 (PMC3834602; doi:10.1038/srep03266)
Supplement: Supplementary Information — for the work [file srep03266-s1.pdf]

Integrated sorting, concentration and real time PCR based detection system for sensitive detection of microorganisms

Monalisha Nayak[1], Deepak Singh[2], Himanshu Singh[1], Rishi kant[1,],Ankur Gupta[1], Shashank Shekhar Pandey[1], Swarnasri Mandal[1,], Gurunath Ramanathan[2*] and Shantanu Bhattacharya[1*]

[1]Department of Mechanical Engineering, Indian Institute of Technology Kanpur, India, [2]Department of Chemistry, Indian Institute of Technology Kanpur, India

Supplementary Information

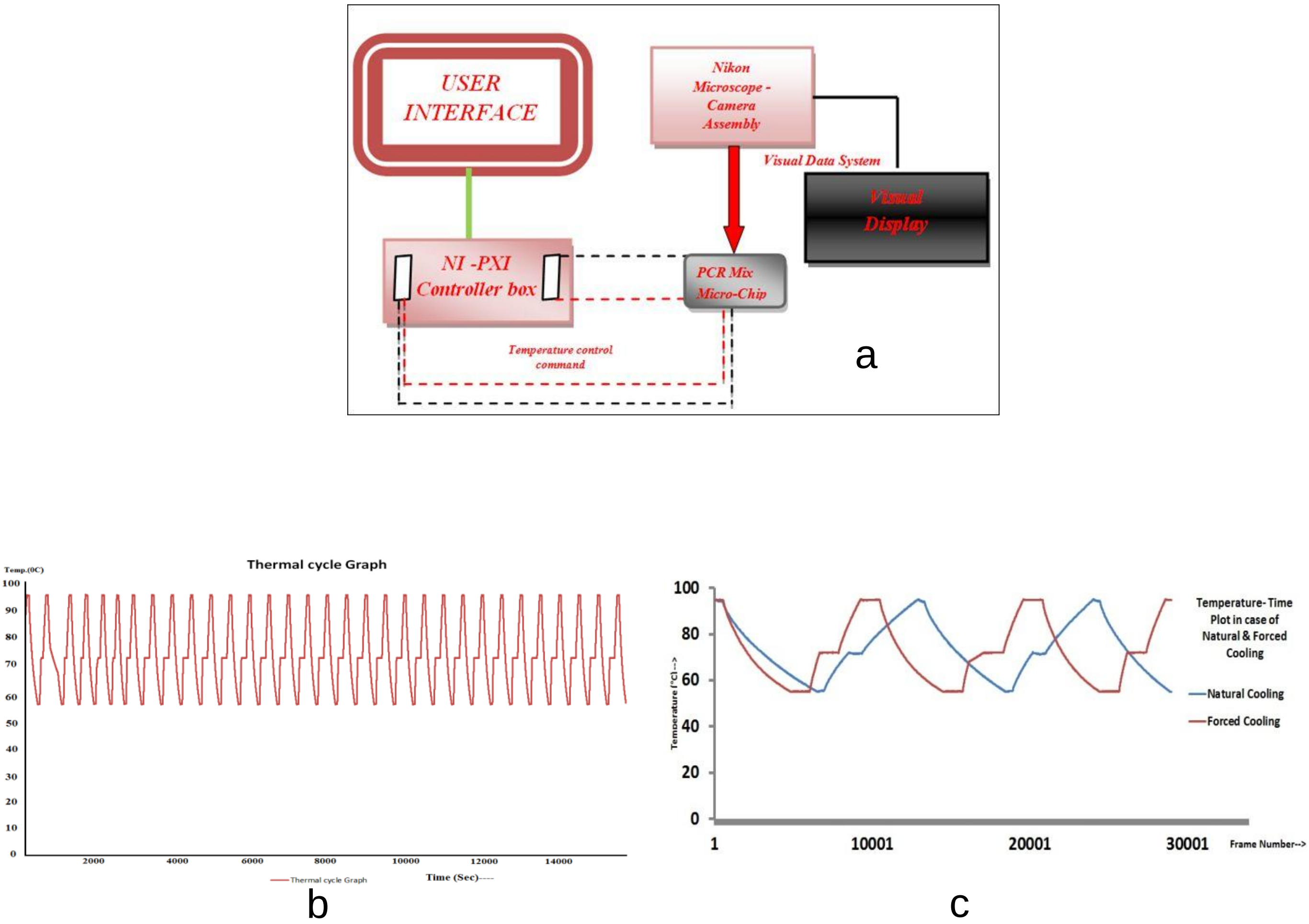

Figure S1:(a) Schematic of instrumentation of thermal cycling (b) Real signal plot of thermal cycling at some point of time within the PCR cycle (c) Comparison between natural and forced cooling.

Sample Concentration

After 0 Minutes

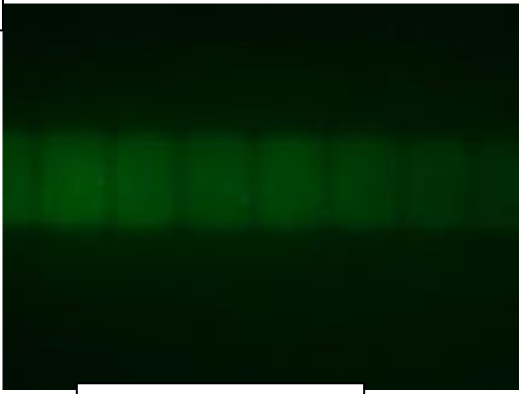

After 15 Minutes

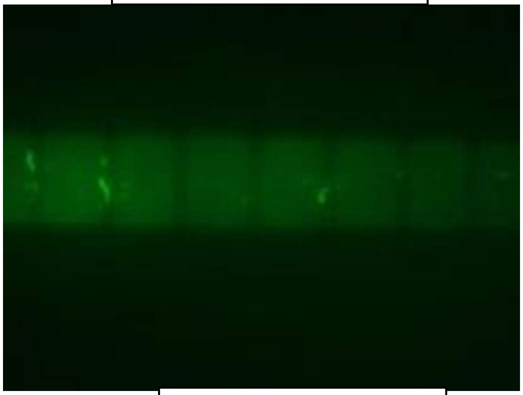

10^9

After 30 Minutes

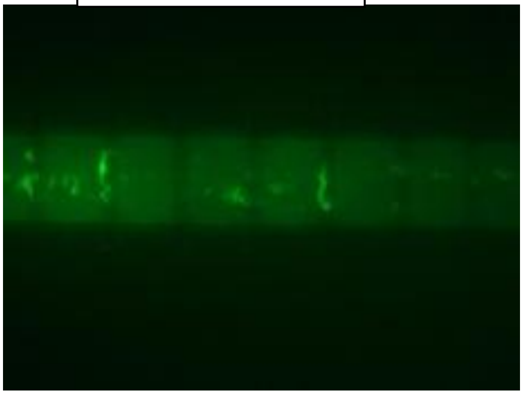

After 45 Minutes

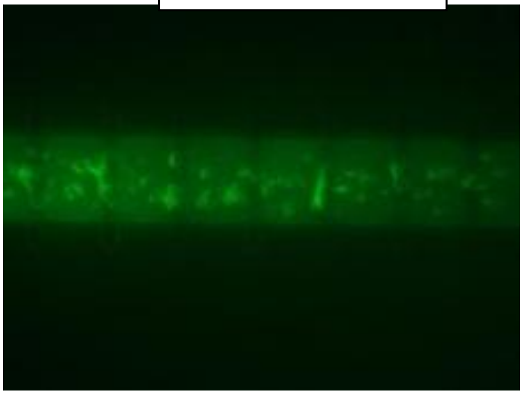

After 0 Minutes

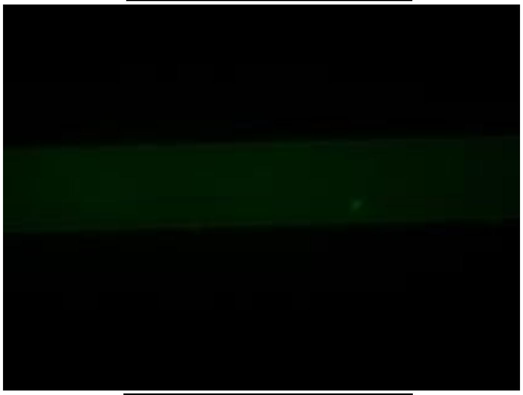

After 15 Minutes

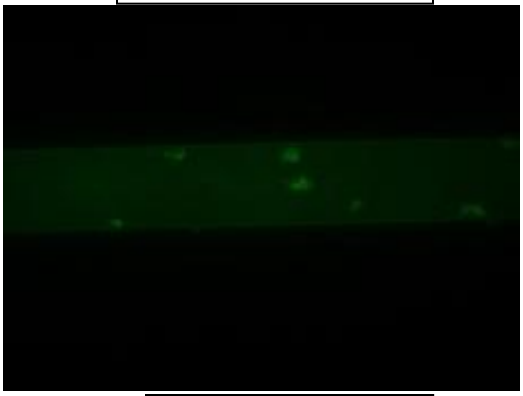

10^7

After 30 Minutes

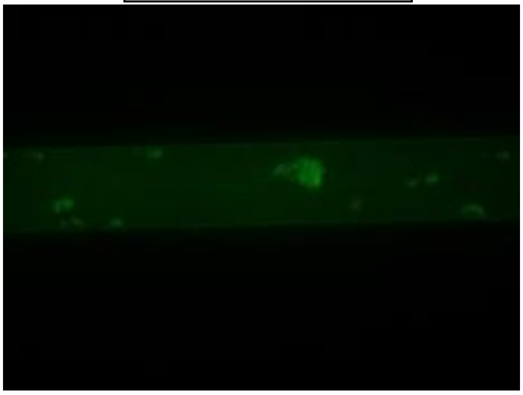

After 45 Minutes

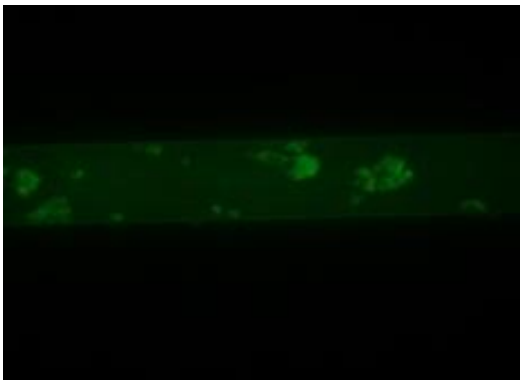

After 0 Minutes

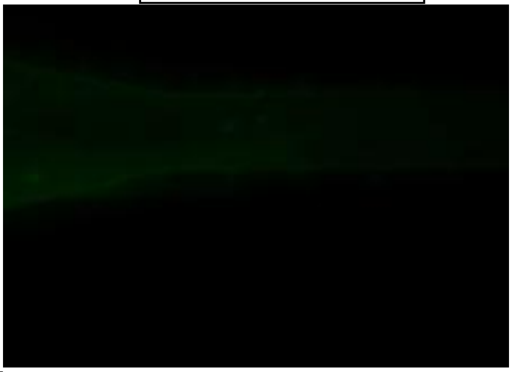

After 30 Minutes

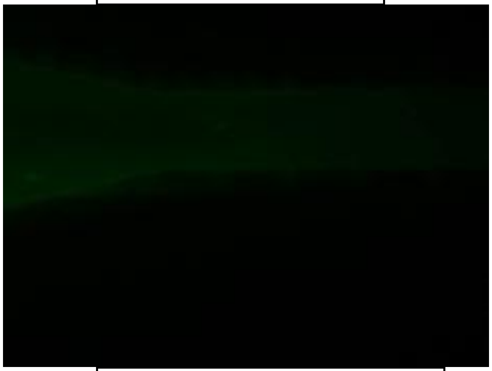

10^6

After 60 Minutes

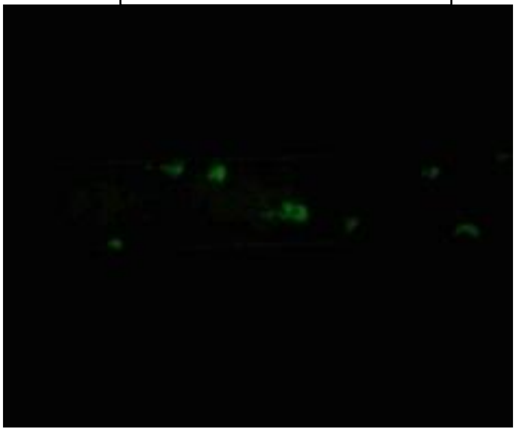

After 120 Minutes

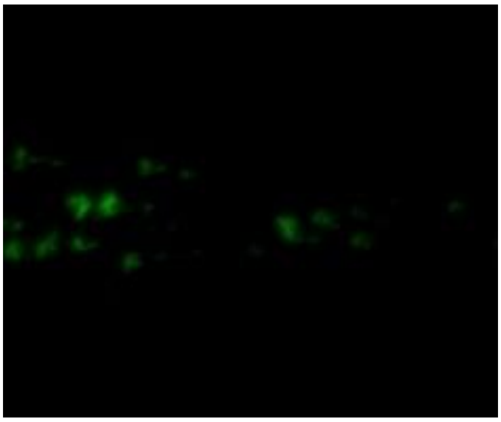

After 0 Minutes

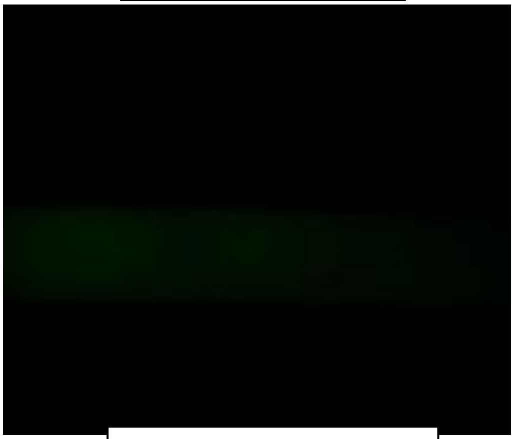

After 30 Minutes

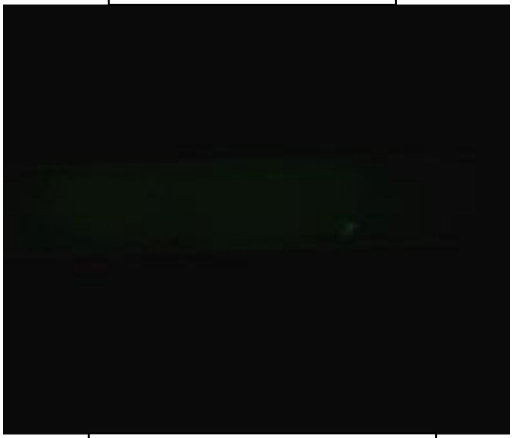

10^4

After 60 Minutes

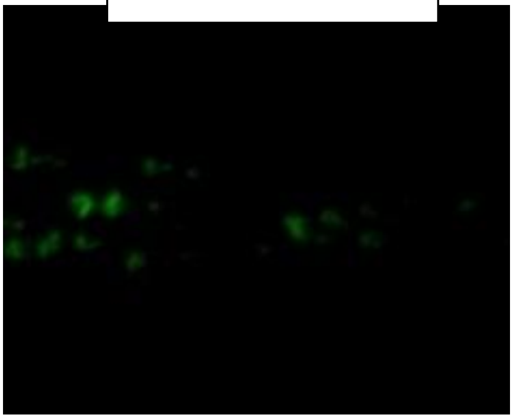

After 120 Minutes

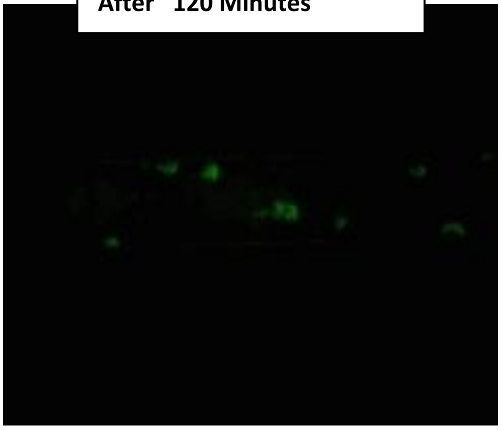

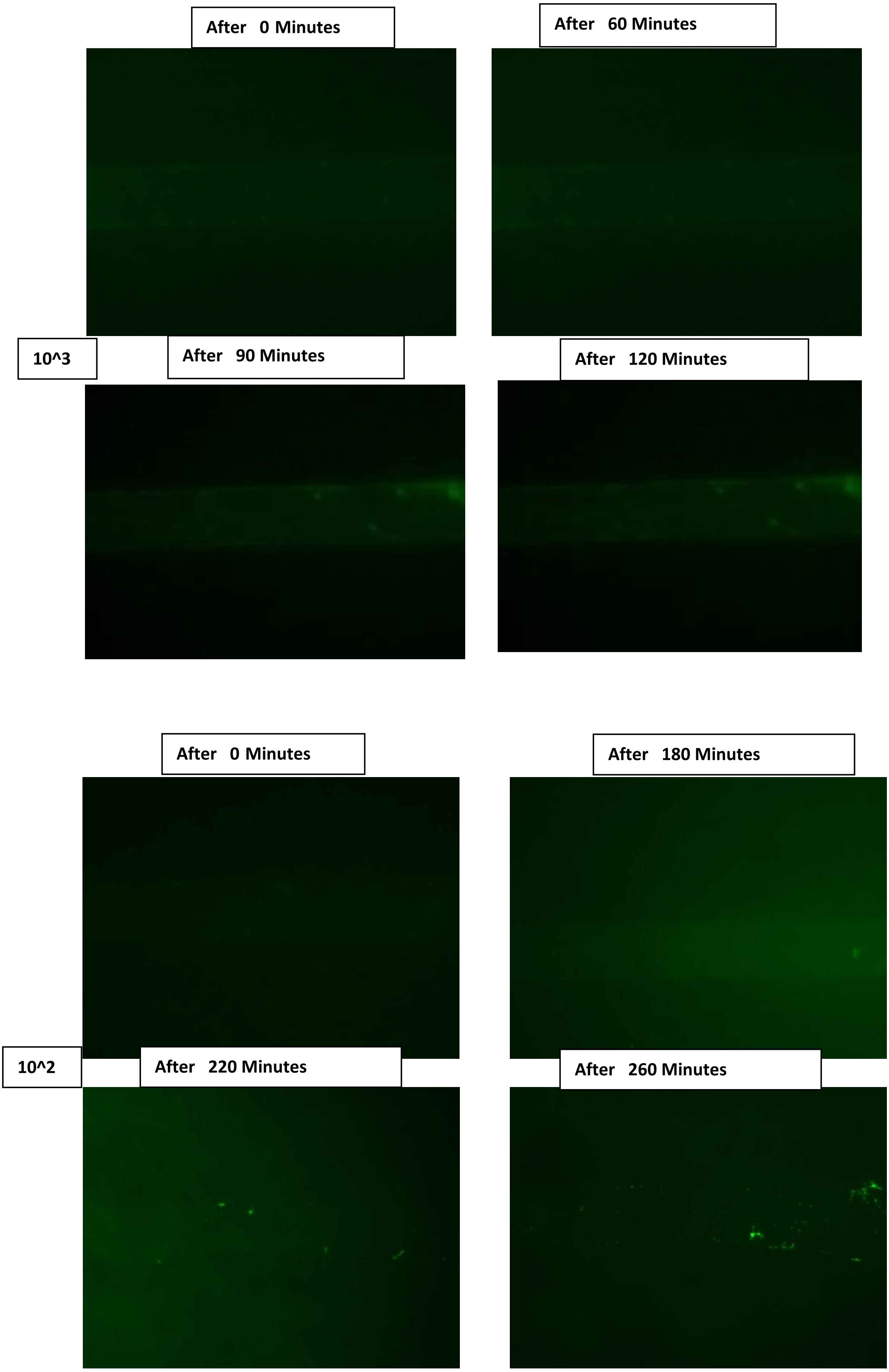

Figure S2: Optical micrographs of cell capture at representative time instances.

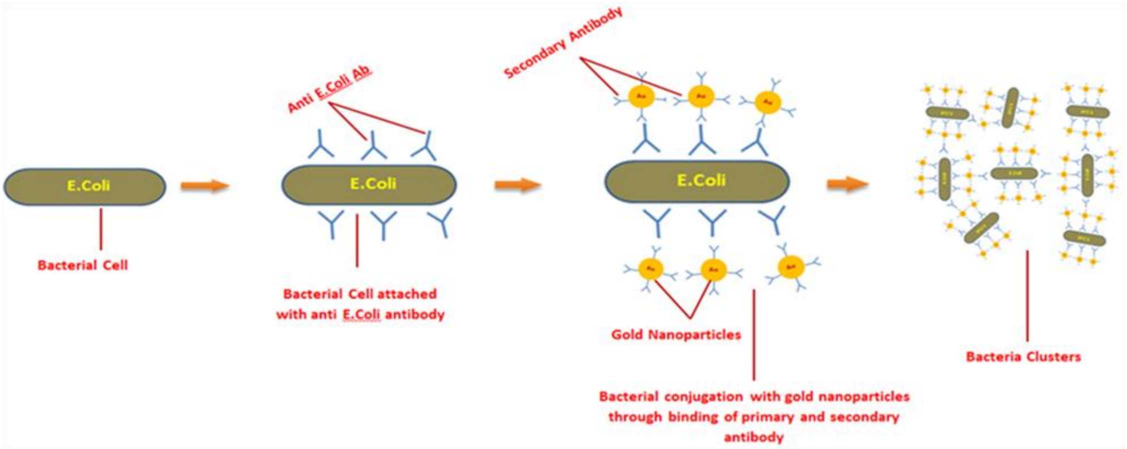

Figure S3:A schematic representation of nano-conjugation process.

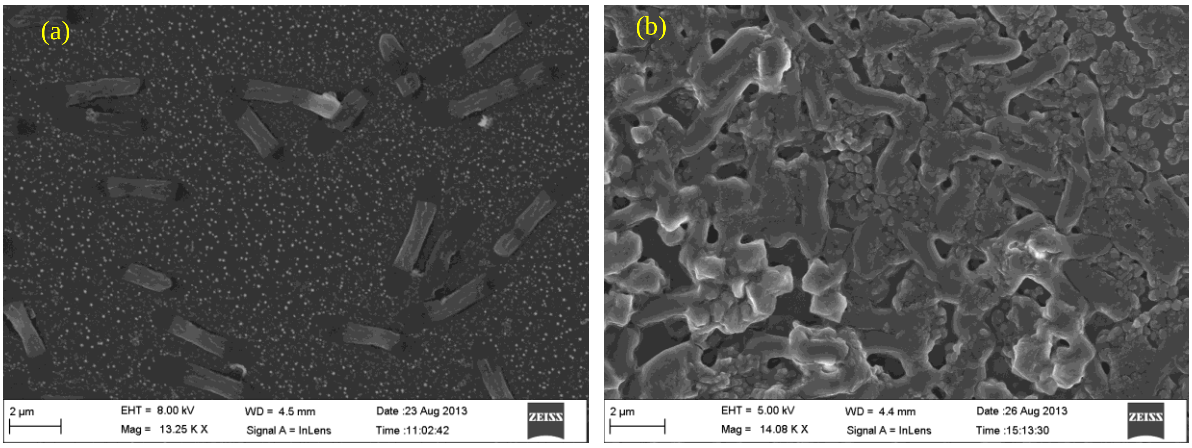

Figure S4 : SEM Images of (a) Normal bacterial cells, (b) Conjugated bacterial cells.

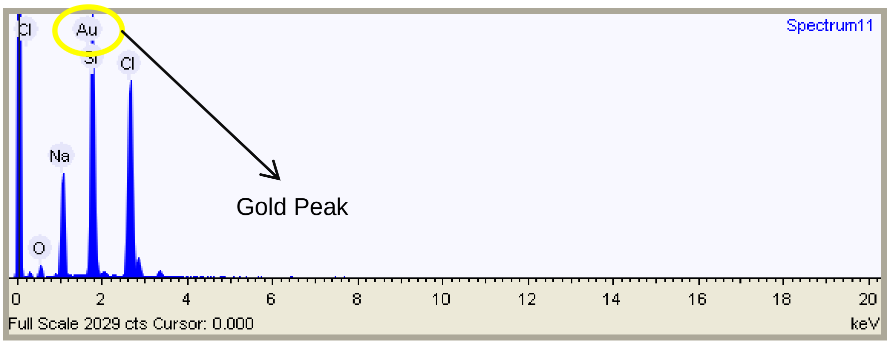

Figure S5 : EDX Spectrum showing the presence of gold nano particles in the nano conjugated bacterial cells(*E.coli*DH5α).

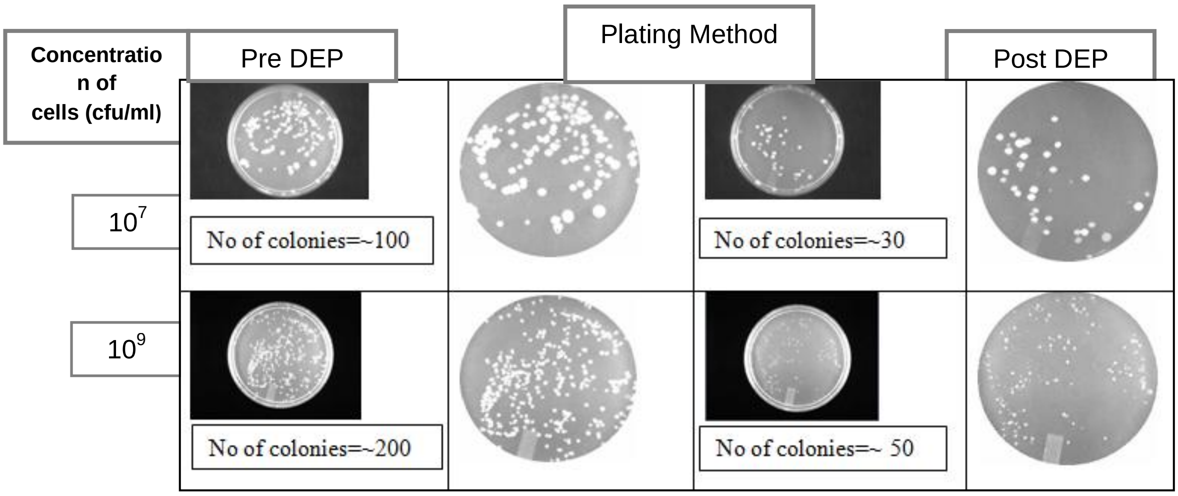

Figure S6:Capturing efficiency testing by plating method of bacterial cells.

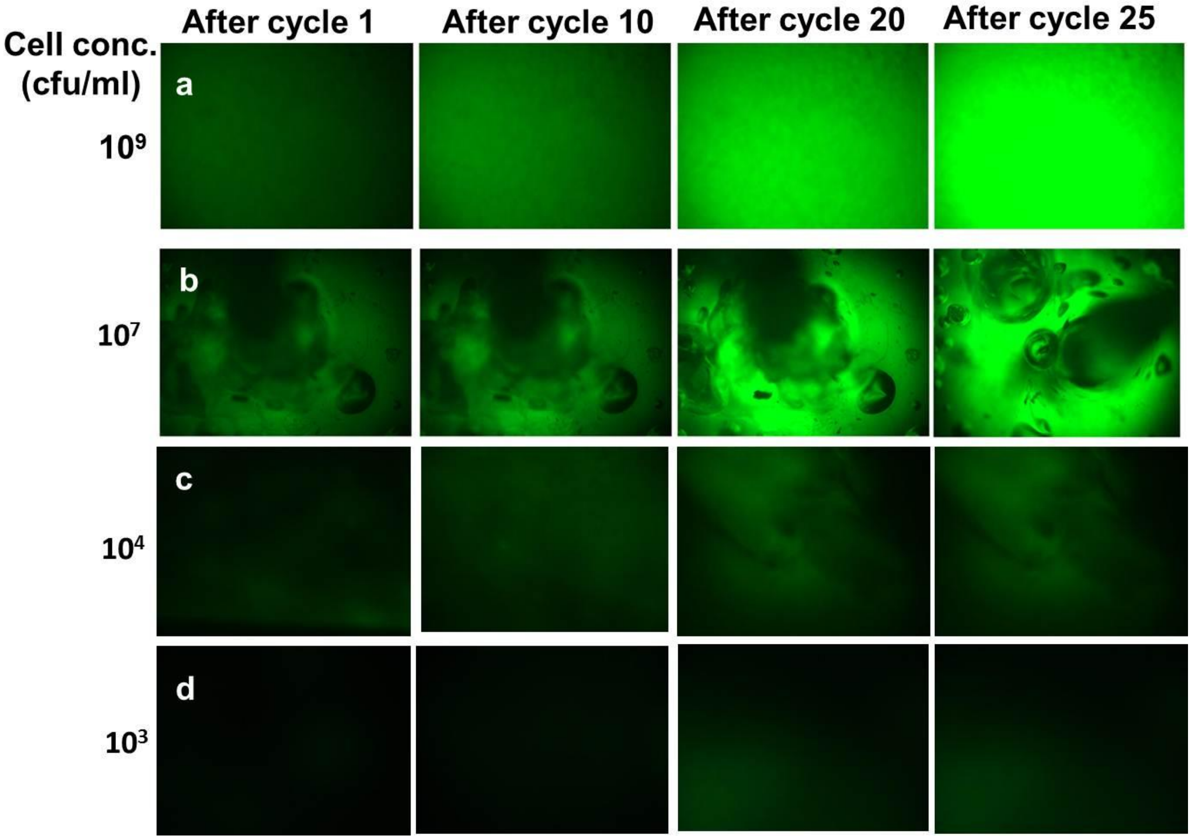

Figure S7: Real time snaps taken during real time PCR after 72°C (a) cell conc. 10^9cfu/ml (b)10^7cfu/ml (c)10^4cfu/ml(d) 10^3cfu/ml .

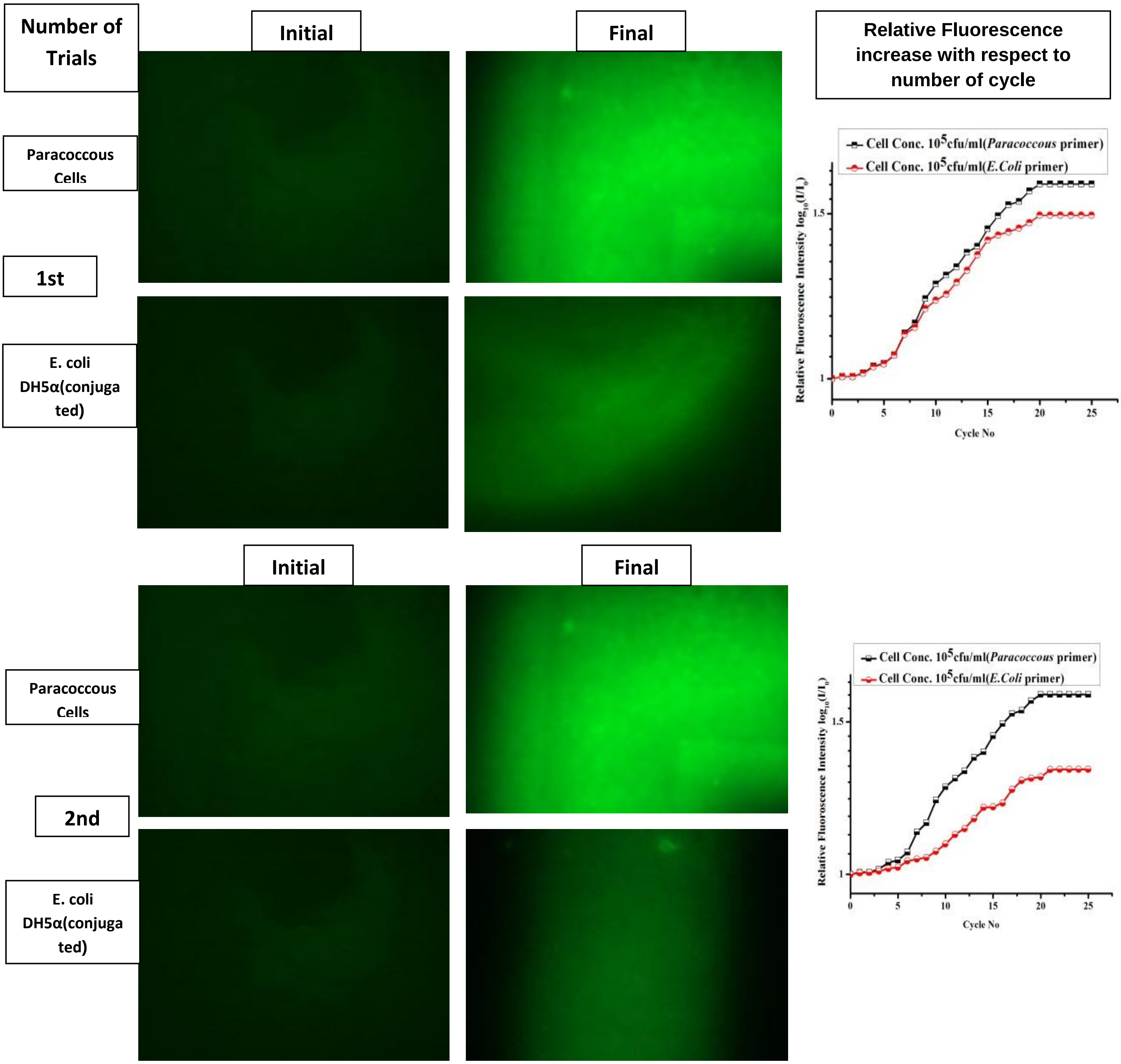

Number of Trials
Initial
Final
Relative Fluorescence increase with respect to number of cycle
Paracoccous Cells
1st
E. coli DH5α(conjugated)
Cell Conc. 10^5cfu/ml(Paracoccous primer)
Cell Conc. 10^5cfu/ml(E.Coli primer)
Relative Fluoroscence Intensity log10(I/I0)
Cycle No
Initial
Final
Paracoccous Cells
2nd
E. coli DH5α(conjugated)
Cell Conc. 10^5cfu/ml(Paracoccous primer)
Cell Conc. 10^5cfu/ml(E.Coli primer)
Relative Fluoroscence Intensity log10(I/I0)
Cycle No

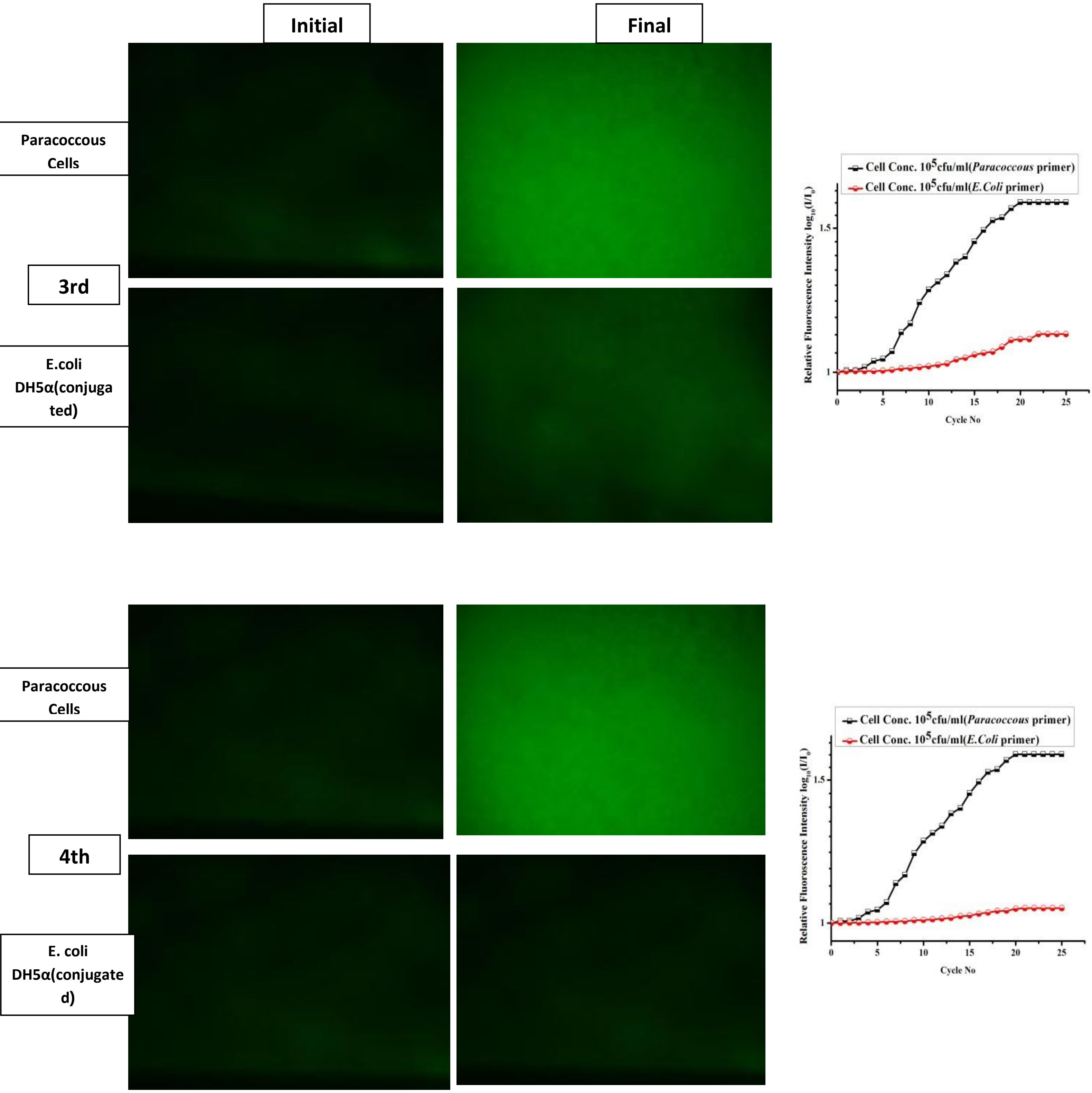

Figure S8: Optical micrographs obtained from RT-PCR of flow through solution after DEP and quantitative estimation of relative fluorescence increase in logarithm scale.

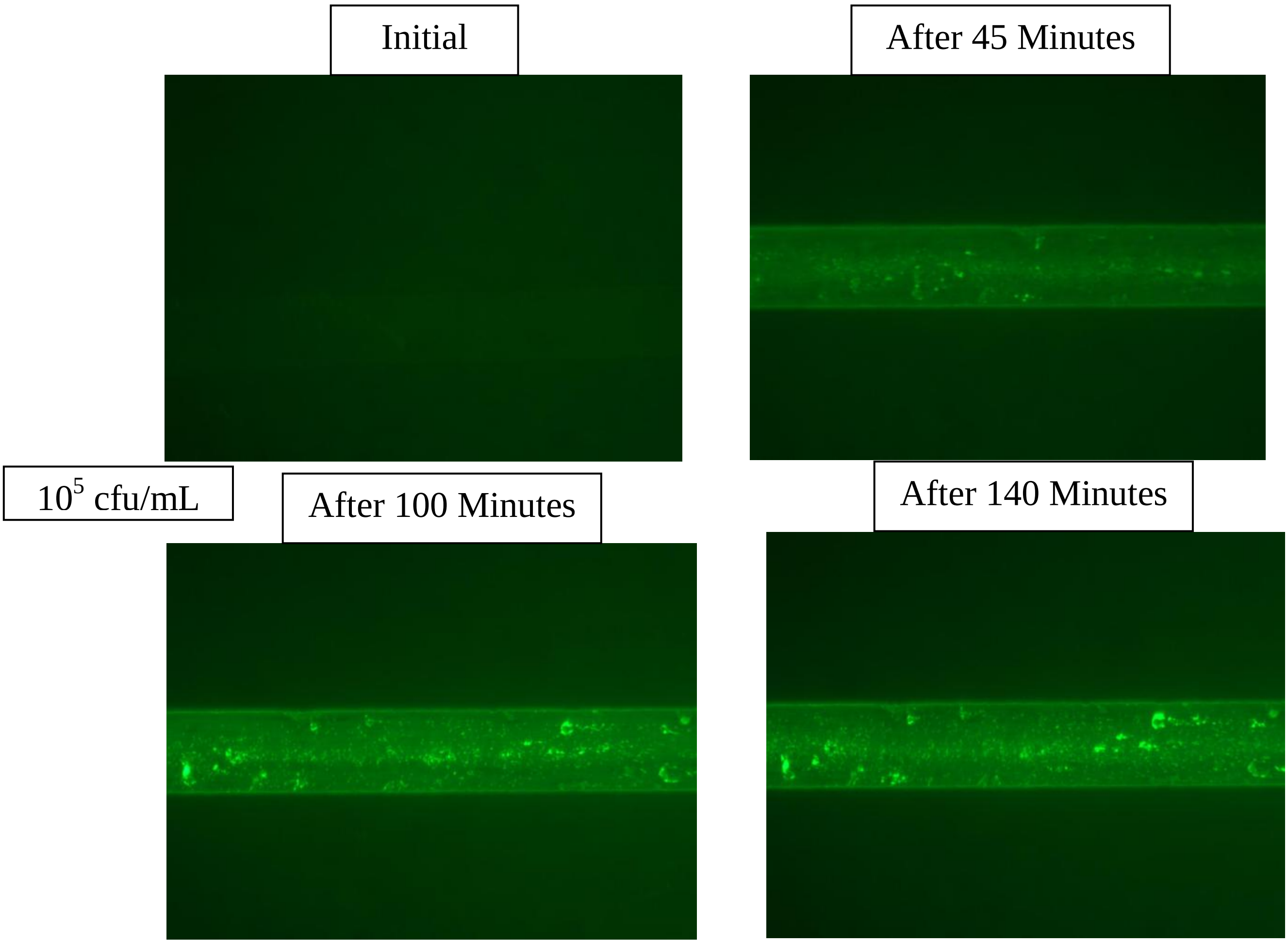

Figure S9: Optical micrographs of cell capture (Juice Sample) at representative time instances.

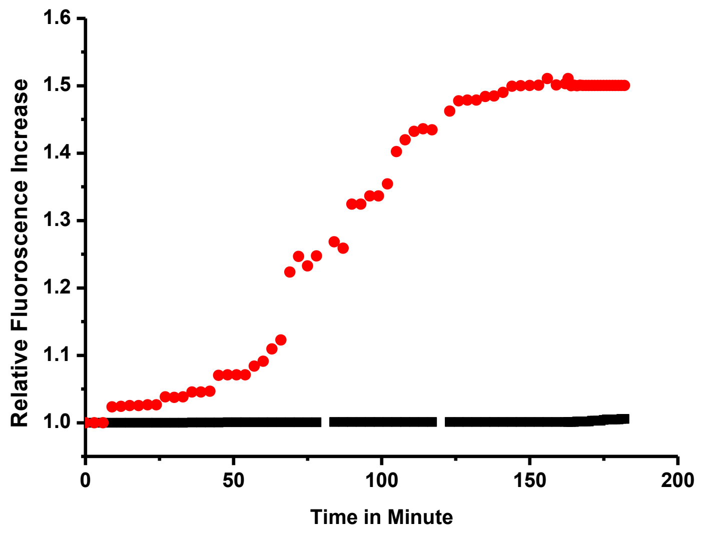

Figure S10: Plot showing the trend in increase in fluorescence intensity during DEP of bacterial Cells (Fruit Juice Sample).

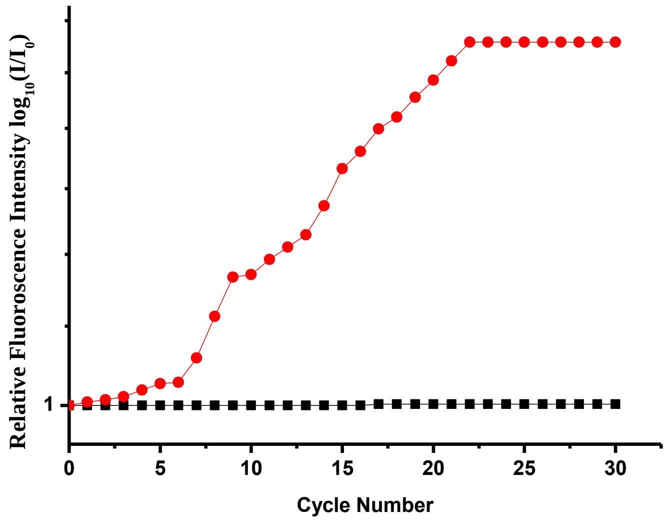

Figure S11: Plot showing the trend in increase in fluorescence intensity during Real time PCR of Captured bacterial Cells (Fruit Juice Sample).
